# Supplementary material for: Time‑resolved multi-omic analysis of paclitaxel exposure in human iPSC‑derived sensory neurons unveils mechanisms of chemotherapy‑induced peripheral neuropathy
Source: Cell Death Dis. 2026 Feb 10;17(1):211. doi: 10.1038/s41419-026-08445-2 (PMC12921266; doi:10.1038/s41419-026-08445-2)
Supplement: Supplementary file 15 — Dataset 1. [file 41419_2026_8445_MOESM15_ESM.zip › CDD_Open_Data/05_Protein/01_Volcano_Plots/VolcanoAndranik/prot_rna_comparison_BH_202306.pdf]

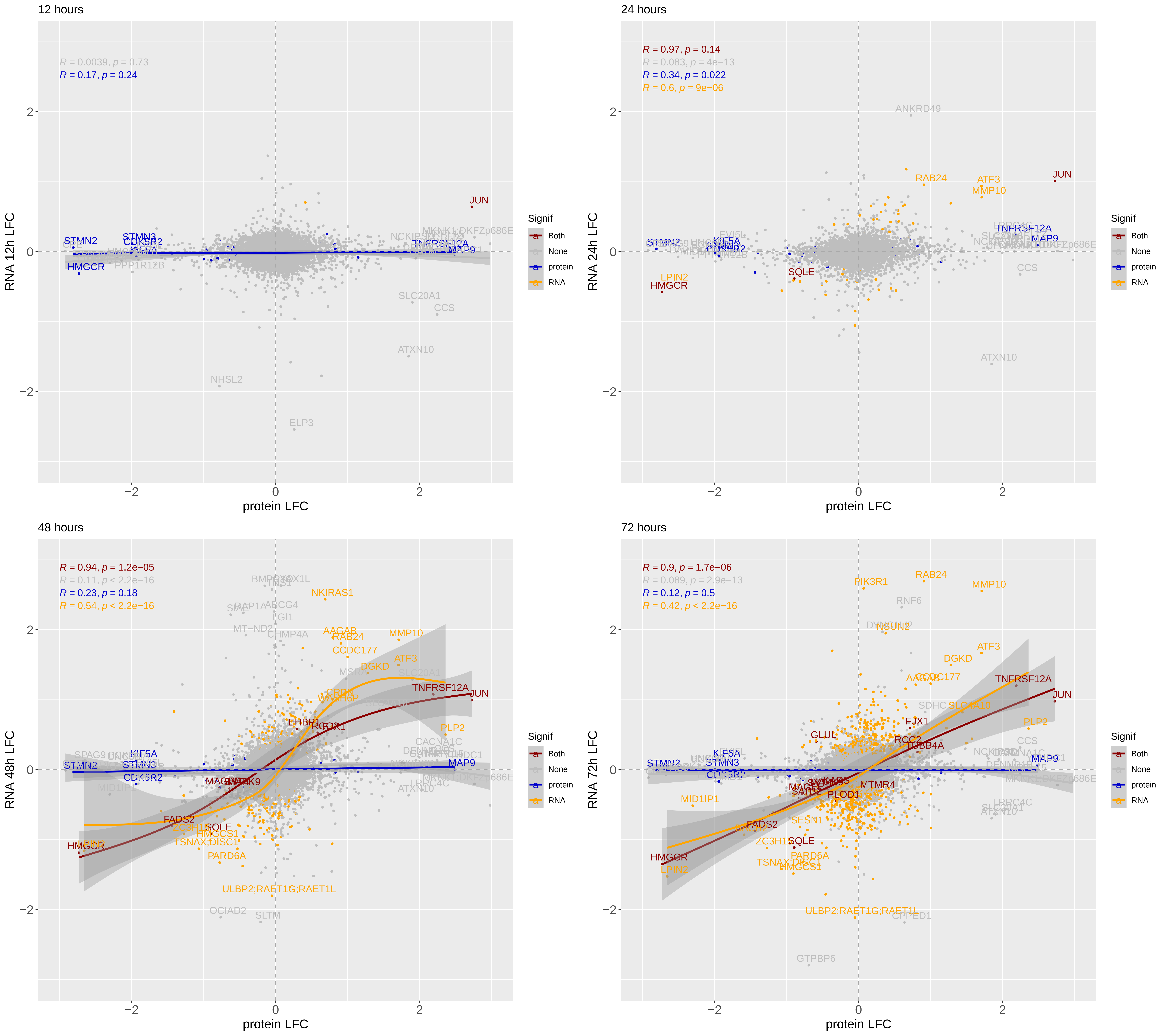

12 hours
R = 0.0039, p = 0.73
R = 0.17, p = 0.24
JUN
STMN2
STMN3
CDK5R2
KIF5A
HMGCR
PPP1R12B
TNFRSF12A
MAP9
SLC20A1
CCS
ATXN10
NHSL2
ELP3
RNA 12h LFC
protein LFC
24 hours
R = 0.97, p = 0.14
R = 0.083, p = 4e−13
R = 0.34, p = 0.022
R = 0.6, p = 9e−06
ANKRD49
RAB24
ATF3
MMP10
JUN
TNFRSF12A
MAP9
STMN2
KIF5A
CDK5R2
LPIN2
HMGCR
SQLE
CCS
ATXN10
RNA 24h LFC
protein LFC
48 hours
R = 0.94, p = 1.2e−05
R = 0.11, p < 2.2e−16
R = 0.23, p = 0.18
R = 0.54, p < 2.2e−16
NKIRAS1
LGI1
MT−ND2
CHMP4A
AAGAB
RAB24
MMP10
CCDC177
ATF3
DGKD
SLC20A1
TNFRSF12A
JUN
EHBP1
PLP2
CACNA1C
KIF5A
STMN2
STMN3
CDK5R2
MAP9
ATXN10
FADS2
SQLE
HMGCS1
PARD6A
HMGCR
ULBP2;RAET1G;RAET1L
OCIAD2
SLTM
RNA 48h LFC
protein LFC
72 hours
R = 0.9, p = 1.7e−06
R = 0.089, p = 2.9e−13
R = 0.12, p = 0.5
R = 0.42, p < 2.2e−16
PIK3R1
RAB24
MMP10
RNF6
NSUN2
ATF3
DGKD
CCDC177
TNFRSF12A
JUN
SDHC
FJX1
GLUL
RCC2
TUBB4A
PLP2
CCS
KIF5A
STMN2
STMN3
CDK5R2
MAP9
MID1IP1
SATB2
PLOD1
MTMR4
SESN1
FADS2
ZC3H15
SQLE
PARD6A
HMGCS1
HMGCR
LPIN2
ULBP2;RAET1G;RAET1L
GTPBP6
RNA 72h LFC
protein LFC
Signif
Both
None
protein
RNA
2
0
−2
